# *In vitro* and *in vivo* antivirus activity of an anti-programmed death-ligand 1 (PD-L1) rat-bovine chimeric antibody against bovine leukemia virus infection

**DOI:** 10.1371/journal.pone.0174916

**Published:** 2017-04-26

**Authors:** Asami Nishimori, Satoru Konnai, Tomohiro Okagawa, Naoya Maekawa, Ryoyo Ikebuchi, Shinya Goto, Yamato Sajiki, Yasuhiko Suzuki, Junko Kohara, Satoshi Ogasawara, Yukinari Kato, Shiro Murata, Kazuhiko Ohashi

**Affiliations:** 1 Department of Disease Control, Graduate School of Veterinary Medicine, Hokkaido University, Sapporo, Japan; 2 Division of Bioresources, Research Center for Zoonosis Control, Hokkaido University, Sapporo, Japan; 3 Global Station for Zoonosis Control, Global Institution for Collaborative Research and Education (GI-CoRE), Hokkaido University, Sapporo, Japan; 4 Animal Research Center, Agricultural Research Department, Hokkaido Research Organization, Shintoku, Japan; 5 Tohoku University Graduate School of Medicine, Sendai, Japan; Emory University School of Medicine, UNITED STATES

## Abstract

Programmed death-1 (PD-1), an immunoinhibitory receptor on T cells, is known to be involved in immune evasion through its binding to PD-ligand 1 (PD-L1) in many chronic diseases. We previously found that PD-L1 expression was upregulated in cattle infected with bovine leukemia virus (BLV) and that an antibody that blocked the PD-1/PD-L1 interaction reactivated T-cell function *in vitro*. Therefore, this study assessed its antivirus activities *in vivo*. First, we inoculated the anti-bovine PD-L1 rat monoclonal antibody 4G12 into a BLV-infected cow. However, this did not induce T-cell proliferation or reduction of BLV provirus loads during the test period, and only bound to circulating IgM^+^ B cells until one week post-inoculation. We hypothesized that this lack of *in vivo* effects was due to its lower stability in cattle and so established an anti-PD-L1 rat-bovine chimeric antibody (Boch4G12). Boch4G12 was able to bind specifically with bovine PD-L1, interrupt the PD-1/PD-L1 interaction, and activate the immune response in both healthy and BLV-infected cattle *in vitro*. Therefore, we experimentally infected a healthy calf with BLV and inoculated it intravenously with 1 mg/kg of Boch4G12 once it reached the aleukemic (AL) stage. Cultivation of peripheral blood mononuclear cells (PBMCs) isolated from the tested calf indicated that the proliferation of CD4^+^ T cells was increased by Boch4G12 inoculation, while BLV provirus loads were significantly reduced, clearly demonstrating that this treatment induced antivirus activities. Therefore, further studies using a large number of animals are required to support its efficacy for clinical application.

## Introduction

Programmed death-1 (PD-1) is an immunoinhibitory receptor that is expressed on activated T cells and B cells and induces immune suppression by binding to PD-ligand 1 (PD-L1) [1]. The PD-1/PD-L1 interaction normally works as a negative feedback system inhibiting excessive immune response, with PD-1 knockout mice developing autoimmune diseases such as lupus-like arthritis and glomerulonephritis [2]. However, this mechanism is often exploited by various chronic diseases to avoid immune elimination. For instance, the expression of PD-L1 has been reported in many human cancers, including melanoma, squamous cell carcinoma, urothelial carcinoma, and solid tumors in the lung, ovary, kidney, pancreas, stomach, and colon [3–9]. In addition, PD-1 upregulation has been demonstrated in tumor-infiltrating T lymphocytes [10]. Importantly, it has been shown that PD-L1 expression is strongly correlated with a progressive stage and worse prognosis, indicating a relationship with disease outcome [5–9].

PD-1 and PD-L1 also play a key role in the failure to eliminate pathogens during chronic infections. Exhausted T cells in mice that are chronically infected with lymphocytic choriomeningitis virus express high levels of PD-1, explaining the mechanism of T-cell dysfunction in this infection [11]. In addition, upregulation of PD-1 has been reported on CD8^+^ T cells, which are specific to human immunodeficiency virus (HIV), hepatitis B virus (HBV), and human T-cell leukemia virus type 1 (HTLV-1) [12–16]. The PD-1 pathway may also result in insufficient clearance during bacterial infection. For instance, during a *Helicobacter pylori* infection, which causes chronic gastritis, PD-L1 is upregulated on human gastric epithelial cells, suggesting that it contributes to inhibiting the T-cell response against *H*. *pylori* [17]. Thus, the PD-1 pathway appears to be a common mechanism of immune evasion in many chronic diseases.

Antibodies that block PD-1 and PD-L1 have proven effective for restoring immune response against cancer and chronic infections, and so several agents for the blockade of human PD-1 and PD-L1 are currently being clinically developed or made commercially available for cancer therapy [18–20]. One clinical study showed that treatment with anti-PD-L1 antibody leads to the inhibition of metastatic lesion growth in 21% and 26% patients with non-small cell lung cancer and melanoma, respectively [21], while other studies have indicated that treatment with anti-PD-1 or anti-PD-L1 antibodies at a dose of 1.0 mg/kg results in objective responses (41% and 29%, respectively) in melanoma patients [22,23]. The effects of these antibodies are less understood for infectious diseases, but anti-PD-L1 antibodies have been shown to enhance T-cell response *in vitro*, such as cell proliferation and the production of interferon-γ (IFN-γ), interleukin-2 (IL-2), tumor necrosis factor-α (TNF-α) and Granzyme B, during HIV, HBV, and HTLV infection [12–15]. Moreover, a phase II study of the anti-human PD-1 antibody Nivolumab against relapsed or refractory adult T-cell leukemia (ATL) caused by HTLV-1 infection is ongoing in Japan [24]. Thus, blockade of the PD-1 pathway represents a promising immunotherapy against cancer and chronic infections.

However, studies on PD-1 and PD-L1 in the field of veterinary medicine are still limited. The high expression of PD-L1 was observed in oral melanoma, osteosarcoma, renal cell carcinoma and hemangiosarcoma in dogs, but there is no report for feline tumors [25,26]. We previously demonstrated that the PD-1 pathway is involved in disease progression during several cattle chronic infections, such as bovine leukemia virus (BLV) infection, Johne’s disease and anaplasmosis [27–30]. Furthermore, antibody blockade of the PD-1/PD-L1 interaction *in vitro* induces activation of the immune response in these diseased cattle [28–31].

BLV belongs to the family Retroviridae and is closely related to HTLV-1 [32,33]. This virus commonly infects host B cells, inducing persistent lymphocytosis (PL) in nearly 30% of infected cattle after the aleukemic (AL) stage, and less than 5% develop B-cell lymphoma following this, which finally leads to death. Furthermore, BLV infection has been linked to decreased milk production [34,35]. Therefore, since there is no effective vaccine and therapeutic method against BLV infection, the development of a novel control method is required to guarantee a stable supply of livestock products. Given its genetic similarity to HTLV-1 and requirements for an effective control method, BLV infection represents a suitable target for treatment with antibodies that block the PD-1 pathway.

In this study, we first attempted to evaluate the *in vivo* function of anti-bovine PD-L1 rat monoclonal antibody (mAb) in a BLV-infected cow. However, we found that this antibody was not sufficiently stable in cattle to be able to assess its long-term antivirus activity. Therefore, we established a rat-bovine chimeric antibody that was specific to bovine PD-L1 (Boch4G12) and examined its ability *in vitro* to bind to bovine PD-L1, interrupt the PD-1/PD-L1 interaction, and activate an immune response against the BLV antigen. We then conducted an *in vivo* analysis using a BLV-infected calf to evaluate its effects on T-cell proliferation and reduction of the BLV provirus. Our findings indicated that Boch4G12 has potential for immune therapies targeting BLV infection.

## Materials and methods

### Cells

Bovine blood samples were obtained from several farmers and veterinarians, and BLV infection was diagnosed at the Hokkaido University Veterinary Teaching Hospital (Sapporo, Japan), as previously described [28]. Peripheral blood mononuclear cells (PBMCs) from healthy or BLV-positive cattle were purified by density gradient centrifugation using Percoll (GE Healthcare, Little Chalfont, UK) and were cultured in RPMI medium (Invitrogen, Carlsbad, CA, USA) containing 10% heat-inactivated fetal calf serum (Thermo Fisher Scientific, Waltham, MA, USA), 2 mM L-glutamine, 100 U/ml penicillin, and 100 μg/ml streptomycin (Thermo Fisher Scientific).

Chinese hamster ovary (CHO) DG44 cells were kindly provided by Dr. Y. Suzuki (Research Center for Zoonosis Control, Hokkaido University), and cells that stably expressed enhanced green fluorescent protein (EGFP) or bovine PD-L1/EGFP were established in a previous study [36]. In brief, a gene encoding the extracellular domain of bovine PD-L1 was cloned into pEGFP-N2 (Clontech, Mountain View, CA, USA). This plasmid or pEGFP-N2 was then transfected into CHO DG44 cells using Lipofectamine® LTX reagent (Thermo Fisher Scientific) to produce PD-L1/EGFP or EGFP cells, respectively. Cells that stably expressed EGFP or PD-L1/EGFP were selected using G418 (800 μg/ml; Enzo Life Sciences, Farmingdale, NY, USA) and then cloned by limiting dilution. EGFP and PD-L1/EGFP cells were maintained in CD DG44 medium (Thermo Fisher Scientific) supplemented with 4 mM GlutaMAX I (Thermo Fisher Scientific) and 0.18% Pluronic® F-68 (Thermo Fisher Scientific).

### Anti-bovine PD-L1 rat monoclonal antibody

A monoclonal antibody that is specific to bovine PD-L1 (4G12) was generated previously [36]. Briefly, a rat was immunized with soluble PD-L1 recombinant protein emulsified with complete Freund’s adjuvant. Lymphocytes from the iliac lymph node were then fused with myeloma cells 24 h after immunization. Antibodies in supernatants produced by the hybridomas were screened using flow cytometry and those that recognized bovine PD-L1/EGFP cells but not EGFP cells were cloned by limiting dilution. Immunization of the rat and cultivation of the hybridoma were performed at Cell Engineering Corporation (Osaka, Japan). To prepare a large amount of this antibody for inoculating cattle, the hybridoma that produced 4G12 was cultured in Hybridoma-SFM (Thermo Fisher Scientific) for 10 days at the Tohoku University Graduate School of Medicine (Sendai, Japan). Following cultivation, 4G12 was purified using Protein G Sepharose® (GE Healthcare Bio-Sciences, Pittsburgh, PA, USA), according to the manufacturer’s protocol.

### Construction of a plasmid vector encoding Boch4G12

Total RNA was extracted from the hybridoma that produced 4G12 using TRIzol™ Reagent (Thermo Fisher Scientific). To identify the sequence encoding the variable region of 4G12, we performed 5′-rapid amplification of cDNA ends (RACE) using the 5′-RACE System (Thermo Fisher Scientific) with primers specific to the rat immunoglobulin gene (5′-ACA AGG ATT GCA TTC CCT TGG-3′ for rat IgG2a, or 5′-CTC ATT CCT GTT GAA GCT CTT GAC GAC-3′ for rat Ig kappa). The cDNA encoding the immunoglobulin was amplified from the poly(C)-tailed RNA using a poly(G) primer and other specific primers (5′-CTC AAT TTT CTT GTC CAC CTT GGT GC-3′ for rat IgG2a, or 5′-CTC ATT CCT GTT GAA GCT CTT GAC GAC GGG-3′ for rat Ig kappa). Following purification of the PCR amplicons using the FastGene® Gel/PCR Extraction Kit (Nippon Genetics, Tokyo, Japan), we cloned them into the pGEM®-T Easy vector (Promega, Madison, WI, USA) and sequenced them using the CEQ™ 2000 DNA Analysis System (Beckman Coulter, Fullerton, CA, USA). The design of the primers used in this study has been outlined in a previous report [37].

Genes encoding the variable region of 4G12 heavy chain coupled with a bovine IgG1 (GenBank accession no. X62916) and that of light chain coupled with a bovine Ig lambda (GenBank accession no. X62917) were commercially synthesized in Medical and Biological Laboratories (Nagoya, Japan). Codon in the synthesized genes was optimized in CHO cells. To reduce the antibody-dependent cell-mediated cytotoxicity (ADCC) response, a mutation was introduced into the binding sites of Fcγ receptors as previously described [36,38,39]. The synthesized genes were cloned into the pDC6 vector (kindly provided by Dr. Y. Suzuki) to produce pDC6-Boch4G12.

### Stable expression and purification of Boch4G12

Cells that produced large and stable amounts of Boch4G12 were established using the dihydrofolate reductase (DHFR)/methotrexate (MTX)-gene amplification system in DHFR-deficient CHO DG44 cells. We transfected pDC6-Boch4G12 into CHO DG44 cells and selected them in CD OptiCHO™ AGT™ medium (Thermo Fisher Scientific) supplemented with 4 mM GlutaMAX I. After cultivation for 3 weeks, cells were screened for the stable expression of Boch4G12 using dot blotting and enzyme-linked immunosorbent assay (ELISA) with anti-bovine IgG Fc polyclonal antibody (Rockland Immunochemicals, Pottstown, PA, USA). Ten of the most superior cell populations for producing Boch4G12 were cloned by limiting dilution, and the top clone was then screened from each of 10 populations using the same methods as described above. The cell clones were cultured in CD OptiCHO AGT medium containing 60 nM MTX (Enzo Life Sciences) for gene amplification and re-screened. This procedure was repeated twice with CD OptiCHO AGT medium containing 250 nM or 1 μM MTX, respectively.

Large amounts of Boch4G12 for inoculating cattle were obtained by shake culture of the cell clones in the medium without MTX at 37°C and 125 rpm with 5% CO_2_ for 14 days. The supernatant of the culture medium was then purified using Ab-Capcher ExTra (ProteNova, Tokushima, Japan), according to the manufacturer’s instructions. The efficiency of antibody purification was verified by sodium dodecyl sulfate-polyacrylamide gel electrophoresis (SDS-PAGE) under reducing (with 2-mercaptoethanol; Sigma-Aldrich, St. Louis, MO, USA) and non-reducing conditions. The concentration of Boch4G12 was determined using bovine IgG Fc ELISA, as described above.

### Binding assay to bovine PD-L1

PD-L1/EGFP cells were incubated with 4G12, Boch4G12, rat IgG2a isotype control (R35-95; BD Bioscience, San Jose, CA, USA), or bovine IgG1 isotype control (Bethyl Laboratories, Montgomery, TX, USA) in serial dilution (0.01–10 μg/ml). APC-conjugated anti-rat Ig (Beckman Coulter) or APC-conjugated anti-bovine IgG Fc (Jackson ImmunoResearch, West Grove, PA, USA) were used as secondary antibodies. Binding of the antibodies was detected by FACS Verse™ (BD Biosciences) and FCS Express 4 (De Novo Software, Glendale, CA, USA).

### Blockade of the PD-1/PD-L1 interaction

The production of bovine PD-1 recombinant protein fused with bovine IgG Fc (PD-1-Ig) has been outlined in a previous report [36]. To confirm the blockade activity of Boch4G12 against the PD-1/PD-L1 interaction, PD-L1/EGFP cells were incubated with 4G12, Boch4G12, rat IgG2a isotype control (BD Bioscience), or bovine IgG1 isotype control (Bethyl Laboratories) in serial dilution (0, 0.32, 0.63, 1.25, 2.5, 5, and 10 μg/ml) at 37°C for 15 min. Biotinylated PD-1-Ig (2 μg/ml) was then added to each well without washing and reacted at 37°C for 30 min. After washing twice, the cells were stained by APC-conjugated streptavidin (BioLegend, San Diego, CA, USA) at room temperature for 30 min and binding of PD-1-Ig to PD-L1/EGFP was detected by flow cytometry, as described above.

### T-cell reactivation assay

PBMCs from healthy cattle were labeled with 0.2 μM carboxyfluorescein diacetate succinimidyl ester (CFSE) and incubated with anti-CD3 (MM1A; WSU Monoclonal Antibody Center, Pullman, WA, USA) at 4°C for 30 min, followed by anti-mouse IgG1 MicroBeads (Miltenyi Biotec, Bergisch Gladbach, Germany) at 4°C for 15 min. CD3^+^ T cells were sorted from the PBMCs using an autoMACS® Pro (Miltenyi Biotec), according to the manufacturer’s instructions. To investigate the effects of Boch4G12 on T-cell proliferation, isolated CD3^+^ T cells were stimulated with 1 μg/ml anti-CD3 (MM1A; WSU Monoclonal Antibody Center) and 1 μg/ml anti-CD28 (CC220; Bio-Rad, Hercules, CA, USA), and co-cultured with mitomycin C-treated (10 μg/ml) EGFP or PD-L1/EGFP cells in RPMI medium in the presence or absence of 10 μg/ml IgG from bovine serum (Sigma-Aldrich) and Boch4G12. After 3 days, the cells were harvested and stained by anti-CD4 (CC30; Bio-Rad) which was pre-labeled with Alexa Fluor 647 using Zenon Mouse IgG1 Labeling Kits (Thermo Fisher Scientific), and anti-CD8 (CC63, Bio-Rad) which was pre-labeled using the Lightning-Link PerCp/Cy5.5 Conjugation Kit (Innova Biosciences, Cambridge, UK). Fixable Viability Dye eFluor® 780 (Thermo Fisher Scientific) was used for a live/dead staining. The intensity of CFSE fluorescence was analyzed by flow cytometry, as described above.

To investigate the effects of Boch4G12 on IFN-γ production, PBMCs from BLV-infected cattle were stimulated with heat-inactivated culture supernatants of FLK-BLV cells and cultured in the presence of 10 μg/ml IgG from rat serum (Sigma-Aldrich), 4G12, IgG from bovine serum (Sigma-Aldrich), or Boch4G12 for 6 days. The concentration of IFN-γ in the culture supernatants was measured using ELISA for bovine IFN-γ (Mabtech, Nacka Strand, Sweden), according to the manufacturer’s protocol.

### Inoculation of the antibodies in BLV-infected cattle

To confirm the *in vivo* effects of 4G12 and Boch4G12, two cattle were introduced into an animal facility at the Animal Research Center, Hokkaido Research Organization (Shintoku, Hokkaido, Japan; Table 1) from its experimental farm. A BLV-infected cow (#368, Holstein, female, 538 kg, 31 months old) was inoculated with 530 mg (1 mg/kg) of the purified 4G12 intravenously; and a healthy calf (#15–11, Holstein, male, 267 kg, 7 months old) was experimentally infected with BLV through administration of BLV-positive leukocytes (1.0 × 10^4^ copies of provirus), which we confirmed developed to the AL stage, and was then inoculated with 260 mg (1 mg/kg) of the purified Boch4G12 intravenously 13 weeks later. Peripheral blood samples were collected from both cattle before inoculation and at least once per week after the inoculation.

**Table 1:** Information on cattle inoculated anti-bovine PD-L1 antibodies.

Cattle	#368	#15–11
**Age**	31 months old	7 months old
**Breed**	Holstein	Holstein
**Sex**	female	male
**BLV infection**	naturally infected	experimentally infected
**Body weight**	538 kg	267 kg
**Inoculated antibody**	4G12	Boch4G12
**Inoculation dose**	1 mg/kg, *i*.*v*.	1 mg/kg, *i*.*v*.

The animal experiments in this study were approved by the Ethics Committee of the Graduate School of Veterinary Medicine, Hokkaido University.

### T-cell proliferation against the BLV antigen

PBMCs isolated from the two cattle that were inoculated with the anti-bovine PD-L1 antibodies were labeled with CFSE and cultured for 6 days with heat-inactivated FLK-BLV supernatants in triplicate. Medium only and heat-inactivated supernatants of FLK cells were used as a negative control. Following cultivation, the PBMCs were stained using anti-CD4-Alexa Fluor 647 (CC30; Bio-Rad), anti-CD8-PerCp/Cy5.5 (CC63; Bio-Rad) and anti-IgM-PE/Cy7 (IL-A30; Bio-Rad). CC30 was pre-labeled with Alexa Fluor 647 using Zenon Mouse IgG1 Labeling Kits (Thermo Fisher Scientific), and CC63 and IL-A30 was pre-labeled using the Lightning-Link PerCp/Cy5.5 Conjugation Kit (Innova Biosciences) and the Lightning-Link PE/Cy7 Conjugation Kit (Innova Biosciences), respectively. The cells were immediately analyzed by flow cytometry, as described above.

### PD-L1 occupancy

The binding of 4G12 to bovine PD-L1 on circulating IgM^+^ B cells was investigated to calculate the PD-L1 occupancy of 4G12 following inoculation. PBMCs isolated from #368 were pre-incubated with 10 μg/ml rat IgG2a isotype control (BD Bioscience) or 4G12, and then incubated with APC-conjugated anti-rat Ig (Beckman Coulter) at room temperature for 20 min. The same secondary antibody pre-incubated with IgG from rat serum (Sigma-Aldrich) at 37°C for 15 min was used as an unstained control. After washing twice, anti-IgM-PE/Cy7 (IL-A30; Bio-Rad) was reacted at room temperature for 20 min. The binding of the antibodies was then detected by flow cytometry, as described above. PD-L1 occupancy was estimated as the percentage of the *in vivo* binding (indicated as the number of cells positively stained by rat IgG2a isotype control) occurred at the total available binding sites (indicated as the number of cells positively stained by a saturated concentration of 4G12).

### BLV provirus loads

BLV provirus loads were measured as previously described [40] with some modification. Briefly, genomic DNA was extracted from 1–5×10^6^ PBMCs of the antibody-inoculated cattle using the Wizard® Genomic DNA Purification Kit (Promega). Amplification of the *BLV Tax* gene was performed in a reaction mixture containing 5 μl of 2×Cycleave® PCR Reaction Mix (TaKaRa Bio, Otsu, Japan), 0.5 μl of Probe/Primer Mix for BLV (TaKaRa Bio), 1 μl of a DNA template and 3.5 μl of PCR-grade water (TaKaRa Bio) using a real-time PCR system (LightCycler® 480 system II; Roche Diagnostics, Mannheim, Germany). Serial dilution of the BLV positive control (TaKaRa Bio) was used to generate calibration curves to determine the provirus loads. Each result is expressed as the number of BLV copies per 50 ng of genomic DNA. The concentration of DNA was determined by ultraviolet (UV) absorbance at 280 nm using a NanoDrop 8000 Spectrophotometer (Thermo Fisher Scientific).

### Statistical analysis

Differences between groups were examined for statistical significance using a paired *t*-test or Wilcoxon signed rank test. For multiple group comparisons, one-way analysis of variance (ANOVA) was performed, followed by Tukey’s test or Dunnett’s test. A *P*-value less than 0.05 was considered statistically significant.

## Results

### *In vivo* antivirus activity of the anti-bovine PD-L1 rat antibody

**Fig 1:**
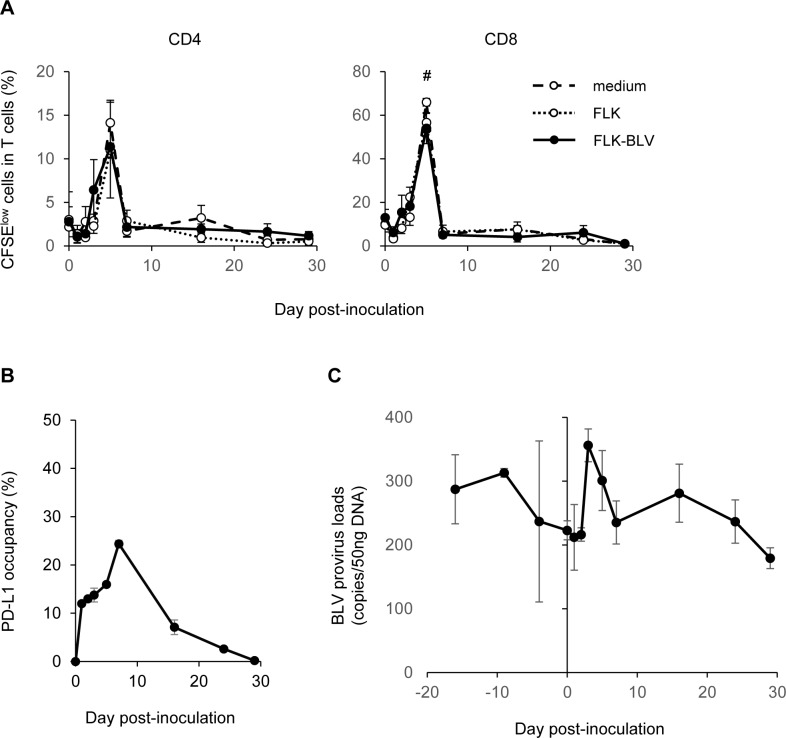
Effects of *in vivo* treatment of a bovine-leukemia virus (BLV)-infected cow with anti-bovine PD-L1 rat monoclonal antibodies. A BLV-infected cow (#368, Holstein, female, 538 kg, 31 months old) was inoculated with 530 mg (1 mg/kg) of the purified 4G12 intravenously. (A) The proliferation of CD4^+^ and CD8^+^ T cells against BLV antigen. Peripheral blood mononuclear cells (PBMCs) isolated from the cow which was inoculated with 4G12 were labeled with carboxyfluorescein diacetate succinimidyl ester (CFSE) and cultured without stimulation (medium) or with the supernatant of FLK or FLK-BLV cells for 6 days. After the cultivation, the proliferation of T cells was immediately analyzed by flow cytometry. A *P*-value less than 0.05 was considered statistically significant. #, *P* <0.05 (FLK-BLV, versus day 0; one-way ANOVA followed by Dunnett’s test). (B) Changes in PD-L1 occupancy on circulating IgM^+^ B cells calculated by the binding of 4G12 to bovine PD-L1. The occupancy was estimated as the percentage of the *in vivo* PD-L1 binding occurred at the total available binding sites. (C) Changes in BLV provirus loads in the cow inoculated with 4G12; the *y*-axis shows the number of BLV copies included in 50-ng DNA extracts of PBMCs. Data are means ± SEM of at least three replicate experiments.

We previously found that the anti-bovine PD-L1 antibody 4G12 induced an immune response *in vitro* [36]. Therefore, to evaluate its antivirus activity *in vivo*, we inoculated a cow (*n* = 1) that had been naturally infected with BLV with 1.0 mg/kg 4G12. T-cell proliferation temporarily increased following inoculation, but this reaction was not BLV-specific (Fig 1A). Furthermore, the PD-L1 occupancy results indicated that the inoculated antibody was only maintained in the blood for one week, following which it dramatically decreased to a level similar to pre-inoculation (Fig 1B). No reduction in BLV provirus loads was observed during the test period (Fig 1C).

### Binding activity of the newly-established Boch4G12

**Fig 2:**
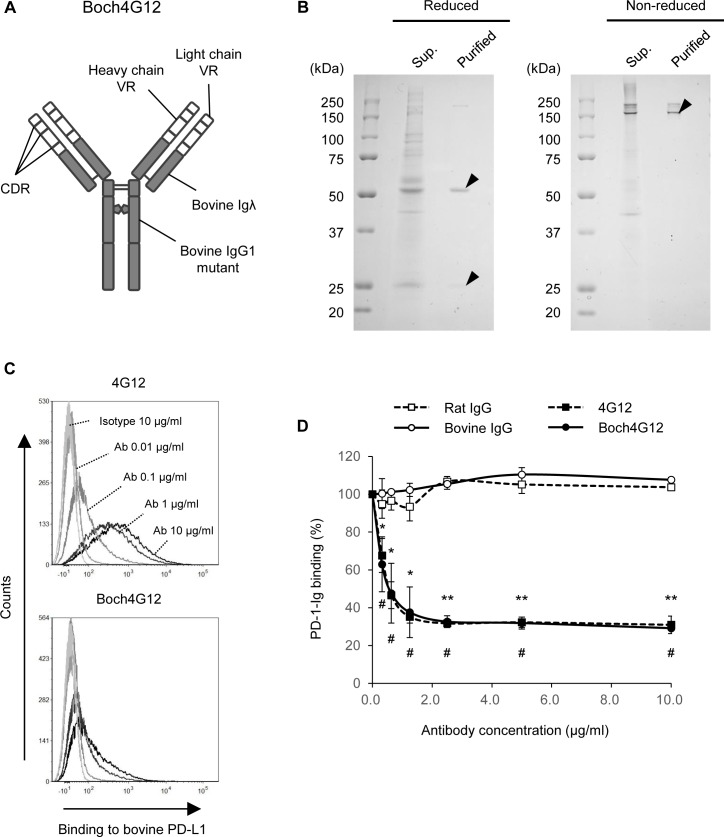
Establishment of Boch4G12 and a binding assay against bovine programmed death-ligand 1 (PD-L1). (A) Diagram of the Boch4G12 structure. White region indicates protein of rat origin and gray indicates protein of bovine origin. VR, variable region; CDR, complementarity-determining region. (B) Confirmation of Boch4G12 expression by SDS-PAGE under reduced and non-reduced conditions. Sup., supernatant before antibody purification; Purified, purified Boch4G12. (C) Binding activity of Boch4G12 against bovine PD-L1, as confirmed by flow cytometry. PD-L1/enhanced green fluorescent protein (EGFP) cells were stained by 4G12 or Boch4G12 at different concentrations (0.01–10 μg/ml) and the antibody binding was detected using anti-rat IgG or anti-bovine IgG secondary antibodies. (D) The blockade activity of Boch4G12 to interrupt the PD-1/PD-L1 interaction assessed using PD-L1/EGFP cells and soluble PD-1 recombinant protein (PD-1-Ig). PD-L1/EGFP cells were pre-incubated with different concentrations of 4G12 or Boch4G12 before being incubated with PD-1-Ig, and the binding of PD-1-Ig was detected by flow cytometry. The *y*-axis shows the rates of PD-1-Ig binding compared with the non-antibody control. A *P*-value less than 0.05 was considered statistically significant. *, *P* <0.05, **, *P* <0.01 (Boch4G12 versus bovine IgG); #, *P* <0.01 (4G12 versus rat IgG), paired *t*-test. Data are the means ± SEM of three replicate experiments.

We hypothesized that the inoculated 4G12 did not show antivirus activities because it was rapidly removed as a xenogenous protein. Thus, a more stable type of antibody (e.g., a chimeric antibody) was required to accurately assess the effects of anti-bovine PD-L1 antibodies. We constructed a plasmid vector encoding Boch4G12, which represented the variable region of the 4G12 heavy chain coupled with a bovine IgG1, and that of the light chain coupled with a bovine Ig lambda (Fig 2A). To avoid cell cytotoxicity, the mutation was introduced into the constant region of Boch4G12, as described previously [36,38,39]. Following transfection of the plasmid vector into CHO DG44 cell lines, we purified Boch4G12 from the culture supernatant. We then confirmed the efficiency of its stable expression and purification using SDS-PAGE, which showed single bands at around 25 kDa representing the light chain and 50 kDa representing the heavy chain under reduced conditions, and 150 kDa representing the whole antibody under non-reduced conditions (Fig 2B).

To examine the *in vitro* functions of Boch4G12, we assessed its binding activity to bovine PD-L1-expressing cells (PD-L1/EGFP cells) by flow cytometry. As with the original antibody, Boch4G12 bound to bovine PD-L1 in a dose-dependent manner (Fig 2C). Since it is essential that therapeutic antibodies targeting PD-L1 molecules interrupt the PD-1/PD-L1 interaction, its blockade activity was confirmed using PD-L1/EGFP cells and soluble bovine PD-1 recombinant protein (PD-1-Ig). Compared with control antibodies that never inhibited the interaction of PD-1-Ig and PD-L1 expressed on the cell membrane, Boch4G12 significantly suppressed the PD-1/PD-L1 interaction in a similar way to 4G12 (Fig 2D). Thus, we successfully established a functional chimeric antibody that is specific to bovine PD-L1.

### *In vitro* effect of Boch4G12- on T-cell function

**Fig 3:**
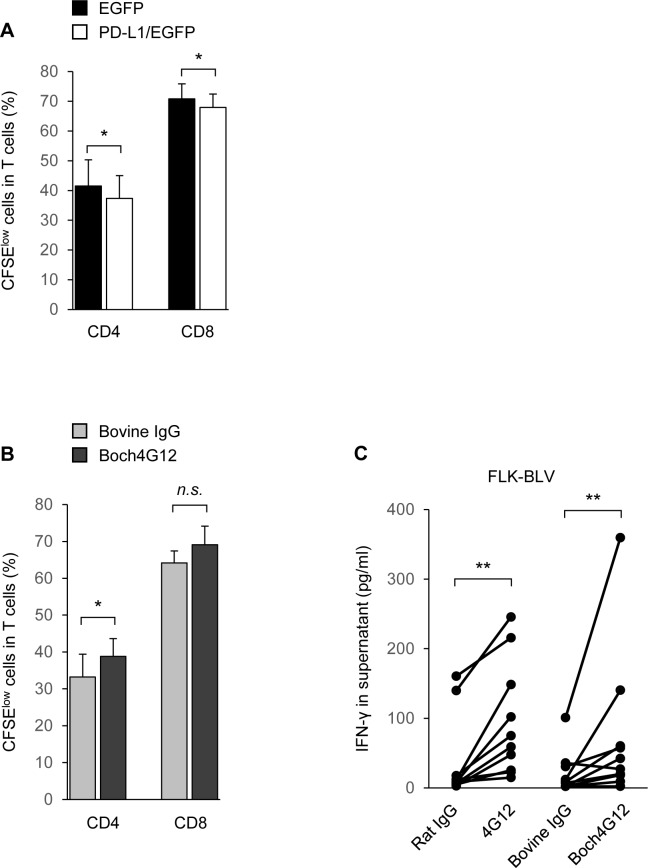
Activation of T-cell proliferation and interferon-γ (IFN-γ) production by Boch4G12. (A and B) The proliferation of CD4^+^ and CD8^+^ cells in purified bovine CD3^+^ T cells. Peripheral blood mononuclear cells (PBMCs) isolated from healthy cattle were labeled with carboxyfluorescein diacetate succinimidyl ester (CFSE), stimulated with 1 μg/ml anti-CD3 and anti-CD28 antibodies, and then cultured for 3 days with enhanced green fluorescent protein (EGFP) cells or programmed death-ligand 1 (PD-L1)/EGFP cells without antibodies (A), or PD-L1/EGFP cells in the presence of 10 μg/ml bovine IgG control or Boch4G12 (B). *n* = 10 and 6, respectively; A *P*-value less than 0.05 was considered statistically significant. *, *P* <0.05, paired *t*-test. (C) IFN-γ production in the culture supernatant of PBMCs from bovine leukemia virus (BLV)-infected cattle (*n* = 10, AL:5, PL:5). PBMCs were stimulated with the supernatant of FLK-BLV cells and cultured for 6 days in the presence of 10 μg/ml rat IgG control, 4G12, bovine IgG control or Boch4G12. The concentration of IFN-γ in the culture supernatants was measured using ELISA. A *P*-value less than 0.05 was considered statistically significant. **, *P* <0.01, Wilcoxon signed rank test. Data are the means of three replicate experiments.

Anti-PD-L1 antibodies exert their function by suppressing the PD-1 signal following PD-L1 blockade. To investigate whether our newly-established Boch4G12 antibody also affected T-cell activation, we isolated CD3^+^ T cells from the PBMCs of healthy cattle and stimulated them with the anti-CD3 and anti-CD28 mAbs. The cells were then cultured with EGFP or PD-L1/EGFP cells in the presence or absence of Boch4G12 for 3 days. We found that the existence of PD-L1/EGFP cells significantly inhibited the proliferation of CD4^+^ and CD8^+^ T cells, and the addition of Boch4G12 suppressed this inhibition in CD4^+^ T cells, but not CD8^+^ T cells (Fig 3A and 3B). These results indicate that the interaction between bovine PD-1 and PD-L1 induces T-cell suppression and that Boch4G12 can restored their proliferation through PD-L1 blockade.

Previous studies have shown that antibodies that bind to bovine PD-L1 upregulate IFN-γ production in PBMCs from healthy or BLV-infected cattle that are cultured in the presence of these antibodies [28,36]. To confirm this effect, we isolated PBMCs from BLV-infected cattle, stimulated them with FLK-BLV supernatant, and then cultured them in the presence of 4G12 or Boch4G12. Both antibodies led to significant upregulation of IFN-γ in the supernatants, compared with the control IgG (Fig 3C), indicating that both the original antibody and Boch4G12 can activate T-cell function in BLV-infected cattle by increasing IFN-γ production. Although Boch4G12 looked like being made significant by the one outlier, the effect of Boch4G12 on IFN-γ production was still significantly higher than that of the control IgG even if that one animal was excluded (*P* <0.02, data not shown)

### *In vivo* analysis of the antivirus activities of Boch4G12

**Fig 4:**
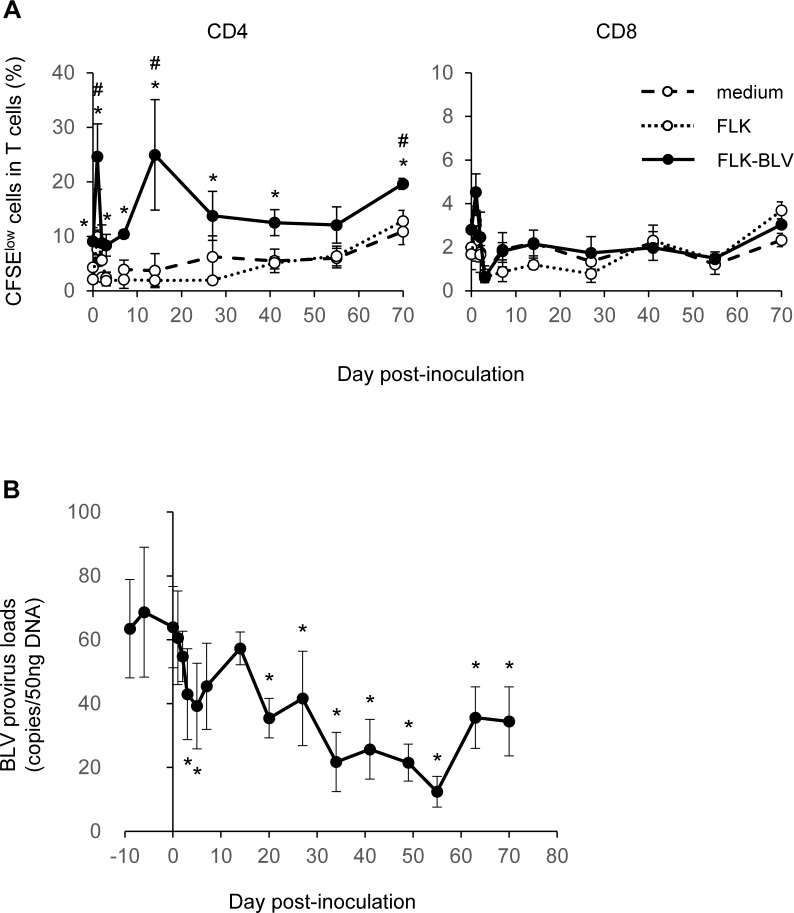
*In vivo* effects of Boch4G12 treatment in a bovine leukemia virus (BLV)-infected calf. A healthy calf (#15–11, Holstein, male, 267 kg, 7 months old) was experimentally infected with BLV through administration of BLV-positive leukocytes and was then inoculated with 260 mg (1 mg/kg) of the purified Boch4G12 intravenously 13 weeks later. (A) The proliferation of CD4^+^ and CD8^+^ T cells against BLV antigen. Peripheral blood mononuclear cells (PBMCs) isolated from the calf which was inoculated with Boch4G12 were labeled with carboxyfluorescein diacetate succinimidyl ester (CFSE) and cultured without stimulation (medium) or with the supernatant of FLK or FLK-BLV cells for 6 days. After the cultivation, the proliferation of T cells was immediately analyzed by flow cytometry. A *P*-value less than 0.05 was considered statistically significant. *, *P* <0.05 (FLK versus FLK BLV; one-way analysis of variance (ANOVA) followed by Tukey’s test), #, *P* <0.05 (FLK-BLV, versus day 0; one-way ANOVA followed by Dunnett’s test). (B) Changes in the BLV provirus loads in the calf inoculated with Boch4G12; the *y*-axis shows the number of BLV copies included in 50-ng DNA extracts of PBMCs. A *P*-value less than 0.05 was considered statistically significant. *, *P* <0.05 (versus day 0; one-way ANOVA followed by Dunnett’s test). Data are means ± SEM of at least three replicate experiments.

Since Boch4G12 exhibited T-cell activation effects *in vitro*, we also examined its *in vivo* function using a BLV-infected calf. A healthy calf (*n* = 1) was experimentally infected with BLV and then inoculated with 1 mg/kg Boch4G12. Blood samples were then collected from this animal to examine T-cell proliferation and BLV provirus loads to evaluate the antivirus activities of this antibody. We found that CD4^+^ T-cell proliferation was upregulated following the antibody inoculation, while CD8^+^ T-cell proliferation was not affected (Fig 4A). Furthermore, the response against the BLV antigen was significantly higher at days 1, 14, and 70 compared with before the inoculation. These results indicate that Boch4G12 prevents PD-1 expressed on BLV-specific T cells from binding to PD-L1, reactivating their immune response. BLV provirus loads in PBMCs were also reduced by up to 74.7% after the inoculation, while they were consistently high before the inoculation (Fig 4B), suggesting that Boch4G12 can induce antivirus activities to decrease provirus loads in BLV-infected cattle. Although the lowest copy number of BLV was observed at day 55, there was no clear association between high T-cell response and low provirus loads at these specific time points. Instead, the reduction of BLV provirus loads occurred following high T-cell response against BLV antigen observed at day 1 and 14 (Fig 4A and 4B).

## Discussion

In this study, we established Boch4G12, a chimeric antibody composed of the variable region from anti-bovine PD-L1 rat mAb (4G12) and the constant region from bovine IgG1. This antibody exhibited similar activities to the original antibody in terms of binding to bovine PD-L1 and interrupting the PD-1/PD-L1 interaction. The inhibition of T-cell function that is induced by PDL1/EGFP cells was restored using Boch4G12, indicating that this antibody has the potential to reactivate exhausted T cells through PD-L1 blockade. In addition, IFN-γ production was upregulated in PBMCs from BLV-infected cattle when cultured with Boch4G12. We previously demonstrated that PD-L1 on IgM^+^ B cells increases in line with the progression of BLV infection [28]. Thus, this observed increase in IFN-γ in the culture supernatant was probably caused by Boch4G12 preventing an interaction between PD-1 on T cells and PD-L1 on infected B cells, reactivating the ability of T cells to respond to the BLV antigen. Finally, a BLV-infected calf that was inoculated with Boch4G12 showed a proliferation of CD4^+^ T cells in response to the BLV antigen and a reduction in BLV provirus loads, suggesting that Boch4G12 has therapeutic potential for controlling BLV infection.

The clinical effects of antibodies that block PD-1 and PD-L1 have mainly been reported in cancer research to date. However, chronic infections such as HIV, HBV, and HTLV-1 are also potential targets of these therapeutic antibodies because PD-1 expression on virus-specific CD8^+^ T cells is upregulated in these diseases and antibody blockade has been shown to induce T-cell activation *in vitro* [12–16]. In addition, the treatment of SIV-infected macaques with anti-PD-1 antibodies results in upregulation of the population of virus-specific CD8^+^ T cells, a reduction in plasma viral loads, and prolonged survival of the animals [41,42]. Moreover, a phase II clinical trial of Nivolumab is currently in progress to assess its effects on relapsed or refractory ATL [24]. Therefore, we considered that BLV infection was an appropriate target for Boch4G12.

We previously demonstrated that the anti-bovine PD-L1 antibodies increase IFN-γ production when bovine PBMCs are cultured in their presence [28,36], and similar results were observed for Boch4G12 (Fig 3C). Though NK cells are a major source of IFN-γ, we did not confirm their role on antivirus activity during the antibody inoculation because compared with T cells, still little is known regarding PD-1 expression on NK cells. According to recent reports, PD-1 signaling inhibits the activating cascade induced by not only the T-cell receptor on T cells but also the activating receptors on NK cells [43], and PD-1 upregulation on NK cells was reported in patients of Kaposi sarcoma and multiple myeloma [44,45]. Moreover, previously we indicated that NK activity, such as production of IFN-γ and perforin, CD69 expression and cytotoxicity, was reduced in cattle in line with BLV provirus loads [46]. Therefore, it will be of great interest to investigate an expression of PD-1 on NK cells and their role for disease development during BLV infection. Then, the *in vitro* activation of T cells induced by Boch4G12 provided a further interest for *in vivo* effects of this antibody. In this study, we inoculated Boch4G12 into a calf that had been experimentally infected with BLV using a similar route to natural infection since it is difficult to assess antivirus activities to a retrovirus *in vitro*. Our results clearly showed that the inoculation of Boch4G12 reduced BLV provirus loads, indicating its antivirus activity *in vivo*, although the number of tested animals was very low.

The CD8^+^ T-cell response plays an important role in the clearance of retroviruses [47,48]. However, Boch4G12 only induced the proliferation of CD4^+^ T cells, not CD8^+^ T cells (Fig 4B). This is probably because the BLV antigen that we used in this study (the culture supernatant of FLK-BLV) represents an exogenous antigen and so is bettered recognized by CD4^+^ T cells. Therefore, further analysis using a BLV peptide presented on MHC class I might help to clarify the effect of Boch4G12 inoculation on CD8^+^ T cells. But this argument is not enough to explain why addition of Boch4G12 did not show improved proliferation in CD8^+^ T cells as alternative modes of antigen presentation, such as cross presentation, could also activate CD8^+^ T cells. Another possibility is that the blockade of PD-1/PD-L1 interaction on CD8^+^ T cells was insufficient to overcome its exhausted status because of other immunoinhibitory molecules like LAG-3, Tim-3 and CTLA-4. Previously, we reported that expression levels of these molecules, LAG-3 [49], Tim-3 [50] and CTLA-4 [51], were upregulated in BLV-infected cattle and that blockades of the interaction between these receptors and their ligands improved immune response *in vitro*, suggesting their important role during BLV infection. In human and mice, it has been demonstrated that the number of immunoinhibitory receptors concurrently expressed on the same CD8^+^ T cells affected the severity of T-cell exhaustion during chronic viral infection [52–54]. Thus, the upregulation of LAG-3, Tim-3 and CTLA-4 also can substantially affect the dysfunction of immune cells in BLV-infected cattle. The study on *Anaplasma marginale* infection in cattle indicated that the percentages of CD4^+^ and CD8^+^ T cells expressing both of PD-1 and LAG-3 were increased during acute phase, suggesting that the PD-1^+^ LAG-3^+^ T cells contributes to the immune deficiency observed in this infection [30]. The coexpression of PD-1 and other immunoinhibitory receptors on CD8^+^ T cells and its role on the severity of exhaustion during BLV infection are currently under analysis, which would help to clarify the cause of less improved proliferation in CD8^+^ T cells.

We found that 4G12 did not show any antivirus activities in cattle, whereas Boch4G12 decreased BLV provirus loads (Figs 1 and 4), despite their same epitope for recognition and same methods for purification and inoculation. One possible explanation for this result is the different infection routes used, i.e., natural versus experimental infection. However, the experimental infection we conducted in this study was imitated the route of natural infection by injection of infected lymphocytes from BLV-infected cattle. Previous study on experimental infections of BLV in cattle indicated that after the infection the provirus loads sharply increased to maximal values at 30–68 days post-inoculation, and then decreased slowly and reached stable states [55]. As 7-month-old calf was inoculated Boch4G12 at 13 weeks post-inoculation, we confirmed that the disease in the experimentally infected calf developed to the AL stage after the acute infection phase (data not shown). Moreover, the provirus loads in both tested animals were categorized as low risk for transmitting BLV to other cattle [40], indicating that the difference in background of the animals had little effect on the results of this study. Another possible explanation is that 4G12 was destabilized by anti-drug antibodies; whereas, Boch4G12 was not since the 7-month-old calf had a less developed immune system. However, several reports showed that immune system of calves were developed until 6 months old [56,57], and modified live vaccines for bovine herpesvirus 1 and bovine viral diarrhea virus are often administered in calves of 3 to 4 months old in the clinical field. Thus, it is less possible that Boch4G12 was reactive because of immature immune response in the 7-month-old calf. The critical difference between the two antibodies was the constant region derived from bovine IgG1 in Boch4G12, which may have led to a high stability in cattle and enhanced the long-term effects to induce an antivirus immune response. Indeed, the reduction in provirus loads continued until day 55, suggesting that inoculated Boch4G12 may have persisted in the calf. In addition, an ADCC-negative mutation had been introduced into the CH2 domain of Boch4G12 based on previous reports [36,38,39]. No information is currently available on the characteristics of the bovine IgG subclass, but this mutation may also contribute to the stability of Boch4G12. One shortfall of this study was the failure to evaluate the occupancy of Boch4G12 in the tested animal, since anti-bovine IgG secondary antibodies are highly cross-reactive to BCR. The detection of circulating Boch4G12 in the serum has less meaning, as shown by the fact that in a phase I study of the anti-human PD-1 antibody, the PD-1 occupancy was maintained at a high level after the circulating antibody had become undetectable [58]. Therefore, antibodies that are specific to the valuable region of Boch4G12 would enable the future evaluation of PD-L1 occupancy of this antibody after inoculation.

BLV infection is spreading globally, including through North America, South America, Asia, and Russia [59–65]. Although most infected cattle are asymptomatic during BLV infection, several studies have shown an association between BLV infection and decreased milk production [34,35]. However, there is currently no effective vaccine and therapeutic method against BLV infection, and so novel methods are required to control this infection. Our finding that inoculation with Boch4G12 decreased BLV provirus loads indicates that this method may represent a novel approach to avoiding the risks of bovine leukemia, particularly in highly-lactating cows and breeding bulls. In conclusion, we succeeded in establishing an anti-bovine PD-L1 rat-bovine chimeric antibody and confirmed its function to reactivate T cells *in vitro* and *in vivo*. Further experiments using larger numbers of cattle are required to support the efficacy of this antibody for clinical application.
